# Long noncoding RNA *CCAT1* rs67085638 SNP contribution to the progression of gastric cancer in a Polish population

**DOI:** 10.1038/s41598-021-94576-9

**Published:** 2021-07-28

**Authors:** Tomasz Olesiński, Anna Lutkowska, Adam Balcerek, Anna Sowińska, P Piotrowski, Tomasz Trzeciak, Tomasz Maj, Piotr Hevelke, Pawel P. Jagodziński

**Affiliations:** 1 Department of Oncological Gastroenterology, Maria Sklodowska-Curie National Research Institute of Oncology, Warsaw, Poland; 2 Department of Biochemistry and Molecular Biology, Poznań University of Medical Sciences, 6 Święcickiego St., 60-781 Poznan, Poland; 3 Department of Computer Science and Statistics, Poznań University of Medical Sciences, Poznan, Poland; 4 Molecular Biology Department, National Institute of Geriatrics, Rheumatology and Rehabilitation, Warsaw, Poland; 5 Department of Orthopedics and Traumatology, Poznan University of Medical Sciences, Poznan, Poland

**Keywords:** Cancer, Risk factors

## Abstract

The role of the long noncoding RNA *CCAT1* NC_000008.10:g.128220661C > T (rs67085638) in the development of colon cancer has been reported. Therefore, we assessed the prevalence of rs67085638 in patients with gastric cancer (GC). We also evaluated the effect of rs67085638 on B-cell-specific Moloney leukaemia virus insertion site 1 (BMI1) transcripts in primary GC and counterpart histopathologically confirmed disease-free margin tissue. Using high-resolution melting analysis, we evaluated rs67085638 frequency in patients with the GC genotype (n = 214) and controls (n = 502) in a Polish Caucasian population. qRT-PCR was used to determine BMI1 transcripts. We observed the trend of rs67085638 association in all patients with GC (*p*_trend_ = 0.028), a strong risk of the GC genotype in male (*p*_trend_ = 0.035) but not female (*p*_trend_ = 0.747) patients, and the association with non-cardia GC (*p*_trend_ = 0.041), tumour stages T3 (*p*_trend_ = 0.014) and T4 (*p*_trend_ = 0.032), differentiation grading G3 (*p*_trend_ = 0.009), lymph node metastasis stage N3 (*p*_trend_ = 0.0005) and metastasis stage M0 (*p*_trend_ = 0.027). We found that significantly increased BMI1 transcripts were associated with the primary GC genotype classified as grade G3 (*p* = 0.011) and as lymph node metastasis N3 (*p* = 0.010) and counterpart marginal tissues (*p* = 0.026, *p* = 0.040, respectively) from carriers of the T/T versus C/C genotypes. rs67085638 may contribute to increased BMI1 transcripts and the progression and rapid growth of GC.

## Introduction

Gastric cancer (GC) is a deadly disease resulting in more than 841,000 deaths each year^1^. The development of GC is due to the interaction of environmental and genetic factors. Environmental carcinogenic factors encompass low socioeconomic status, tobacco smoking, radiation, *Helicobacter pylori* infection, low consumption of fruits and vegetables and high intake of salty and smoked food, male sex, obesity and older age^2–6^. However, not everyone is exposed to the environmental carcinogenic factors that develop into GC, which suggests a strong genetic background contributing to its occurrence^6–8^. A genetic study demonstrated the different variants of genes that contribute to the development of GC^8^. Genome-wide association studies (GWAS) have revealed many loci as major genetic components in the development of GC^9–11^. Many previously studied single nucleotide polymorphisms (SNPs) did not reach statistical significance in GWAS, but they may force the development and progression of GC^6–8^.

Long noncoding RNAs (lncRNAs) are a class of transcripts that are longer than 200 nucleotides in length and are not translated to proteins^12^. In the nuclei and cytosol, lncRNAs play critical roles in various mechanisms regulating gene expression, which can be related to development, progression and drug resistance in cancer^13–15^. Abnormal lncRNA expression has been demonstrated in many malignancies, including GC^15^.

Recently, studies have suggested that colon cancer-associated transcript-1 (CCAT1), also designated LOC100507056 or the cancer-associated region lncRNA-5, plays a role in the growth and invasion of GC^16–19^. *CCAT1* includes a region 2,628 base pairs in length, and its expression results in two isoforms of lncRNAs: CCAT1-L and CCAT1-S. CCAT1-L is exclusively situated in the nucleus, whereas the short isoform, CCAT1-S, is positioned in the cytoplasm^20^.

The *CCAT1* NC_000008.10:g.128220661C > T (rs67085638) polymorphism is associated with the development of colon cancer^21^. However, little is known about the contribution of rs67085638 in the development of GC. In our study, we assessed the prevalence of the *CCAT1* rs67085638 polymorphism in patients with GC in a Polish Caucasian population. We also evaluated the distribution of rs67085638 in different clinicopathological characteristics of GC.

Recent findings demonstrated that the presence of the minor allele of rs67085638 increased the expression of CCAT1^22^. Moreover, lncRNA CCAT1 knockdown significantly downregulated B-cell-specific Moloney leukaemia virus insertion site 1 (BMI1) mRNA in GC^23^. Therefore, we also assessed the effect of *CCAT1* rs67085638 on the transcript levels of BMI1 in primary GC tissue and counterpart histopathologically confirmed disease-free margin tissue.

## Materials and methods

### Study population

The studied subjects included 214 patients with gastric adenocarcinoma (GC, 54 cardia and 160 non-cardia) who were diagnosed by oncologists. All cases were gastric adenocarcinoma and were confirmed by pathological examination with routine evaluation of differentiation stage and grade according to the staging system of the tumour node metastasis (TNM) classification system and the World Health Organization (Table 1)^24^. Patient data and primary GC tissue samples were obtained from subjects enrolled between July 2016 and January 2019 at the Department of Oncological Gastroenterology, Maria Sklodowska-Curie Oncology Center, Warsaw, Poland. The inclusion criteria were age over 18 years, new diagnosis of gastric adenocarcinoma, and ability to endure surgical treatment or chemotherapy. The exclusion criteria encompassed patients with a non-adenocarcinoma and the presence of an extragastric tumour. Subjects with severe systemic disorders were also excluded from the group of patients with GC.

**Table 1:** Available clinicopathological characteristics of patients with gastric cancer.

**Sex** No. of patients	Male 139	Female 75		
^a^**Mean age (years) ± SD**	56.1 ± 11.8	57.5 ± 12.1		
**GC localization** No. of patients	Cardia 54	Non-cardia 160		
**Histological type** No. of patients	Diffuse 111	Intestinal 79	Mixed 18	Undetermined 6
**Tumour stage** No. of patients	T1 24	T2 32	T3 86	T4 72
**Lymph node metastasis stage** No. of patients	N0 78	N1 35	N2 42	N3 59
**Metastasis stage** No. of patients	M0 192	M1 22		
**Histological grading** No. of patients	G1 7	G2 66	G3 141	

*T* tumour, *N* node, *M* metastasis, *G* grade^22^.

^a^Age at first diagnosis.

The control group was matched to patients by age and sex and included 175 healthy females with a mean age of 56.9 ± 12.3 years and 327 healthy males with a mean age of 55. 8 ± 11.8 years randomly selected during routine medical examinations at the Department of Radiotherapy of the Greater Poland Cancer Center in Poznań, Poland, Subjects who were pregnant, had a history of cancer or had blood relatives with GC going back two generations were excluded from the control group. We also obtained body mass index (BMI) data for the controls and patients with GC as well as the presence of diabetes in the GC patients (Supplementary Table 1^25^).

### Tissue samples

We found the greatest association of rs67085638 with G3 differentiation grading and with N3 lymph node metastasis in patients with GC. Therefore primary GC tissue and counterpart histopathologically confirmed disease-free margin tissue from these patients were collected to determine the influence of rs67085638 on the level of BMI1 transcript. Primary GC tissue samples and distal surgical resection margin histopathologically confirmed disease-free tissue samples were obtained from 42 patients with a mean age of 51.3 ± 7.6 years and classified as grade G3 at the time of surgery and from 42 patients with a mean age of 52.4 ± 6.6 years and classified as N3 at the time of surgery at the Department of Oncological Gastroenterology, Maria Sklodowska-Curie Oncology Center, Warsaw. A portion of the tissue sample was immediately snap-frozen in liquid nitrogen and stored at − 80 °C until RNA isolation was performed.

### Genetic analysis

DNA was isolated from peripheral blood cells via a salting-out procedure. The primers were designated using Oligo 7.6 software (DBA Oligo, Inc., Colorado Springs, CO). The NC_000008.10:g.128220661C > T (rs67085638) polymorphism DNA fragment (140 bp) was amplified using the following primers: forward 5′ GCTGTAAATAACGCTGAT 3′ and reverse 5′AACTGAATGAGATGAAGG 3′. The rs67085638 SNP was then genotyped via high-resolution melting (HRM) curve analysis previously described^26^ using HOT FIREPol EvaGreen (Solis BioDyne, Tartu, Estonia) with a LightCycler 480 system (Roche Diagnostics, Mannheim, Germany). The presence of this SNP was reanalysed by Sanger sequencing analysis of arbitrarily chosen samples, comprising 10% of the samples from both cases and controls. The concordance rate between HRM and sequencing was 100%.

### Reverse transcription and quantitative real-time PCR (qRT-PCR) analysis of BMI1 transcript levels in GC tissue and histopathologically confirmed disease-free margin tissue

Frozen primary GC and distal surgical resection counterpart margin histopathologically confirmed disease-free tissues were homogenized, and total RNA was isolated according to the method of Chomczyński and Sacchi^27^. RNA quality was determined spectrophotometrically and by agarose gel electrophoresis. RNA samples were treated with DNase I, quantified, and reverse-transcribed into complimentary DNA (cDNA) (Supplementary Table 2). Quantitative analysis of BMI1 cDNA (Supplementary data 1) was performed by the Light Cycler480 II Real-Time PCR System (Roche Diagnostics GmbH, Mannheim, Germany) with SYBR Green I as the detection dye. BMI1 cDNA was quantified using the relative quantification method with a calibrator (Supplementary Table 2). The quantity of the BMI1 transcript in each sample was standardized by the geometric mean of reference transcript levels: hydroxymethylbilane synthase (HMBS) and beta-2-microglobulin (B2M) (Supplementary Table 2). The BMI1 mRNA levels were expressed as multiples of these cDNA concentrations in the calibrator.

### Statistical analysis

The distinction in genotypic prevalence between the patients and controls and their genotype deviation from Hardy–Weinberg (HW) equilibrium were evaluated using a χ^2^ test. The rs67085638 SNP was tested for association with GC using the Cochran–Armitage *p*-trend test (*p*_trend_). The χ^2^ and Fisher exact tests were used to determine the differences in genotypic and allelic distributions between the patients and controls. The odds ratios (ORs) and 95% confidence intervals (95% CIs) were also calculated. Logistic regression analysis was used to adjust for the effect of age, BMI and the presence of diabetes. A p-value of < 0.05 was considered statistically significant. Statistical analysis comparing BMI1 transcript levels between the T/T versus C/C and C/T versus C/C genotype carriers was evaluated using the Kruskal–Wallis test with Dunn's post hoc test. Statistical analyses were conducted using Statistica version 10, 2011 (Stat Soft, Inc., Tulsa, USA), as previously described^28^.

### Ethical approval

The study procedures were approved by the Local Ethical Committee of the Poznań University of Medical Sciences (reference number of ethical approval: 673/15 and 190/19). The study was carried out in accordance with the approved guidelines. Informed consent was obtained from all individual participants included in the study.

## Results

### Distribution of the *CCAT1* rs67085638 SNP between the group of patients with GC and the control group

The χ^2^ test of HW equilibrium showed values of 0.973 and 0.949 for the patients with GC and the control group, respectively. The genotype distribution of the rs67085638 SNP in the group of patients is presented in Table 2. We found a significant association of the rs67085638 SNP with all the patients with GC, and the *p*-trend value calculated for the rs67085638 polymorphism was *p*_trend_ = 0.028. The logistic regression analysis, which was adjusted for the effects of age, BMI and presence of diabetes, demonstrated an association of the rs6983267 SNP with T/T as well as the T/T + C/T genotype and T allele for all the patients with GC. For the T/T versus C/C genotypes, the adjusted OR was 1.360 (95% CI 1.011–1.828, *p* = 0.041); for the C/T + T/T versus C/C genotypes, the adjusted OR was 1.423 (95% CI 1.025–1.977, *p* = 0.035); and for T versus C, the adjusted OR was 1.373 (95% CI 1.056–1.784, *p* = 0.018). However, there was no significant association of the rs6983267 SNP for C/T versus C/C, where the adjusted OR was 1,430 (95% CI 0.981–2.085, *p* = 0.063).

**Table 2:** Prevalence of the *CCAT1* rs67085638 polymorphism among patients with GC and controls.

Genotype	Patients (frequency)	Controls (frequency)	Odds ratio (95%CI)	*p*^a^	Adjusted odds ratio (95%CI)^b^	*p*	*p*_trend_
**All**							
C/C	85(0.40)	239 (0.48)	Referent		**Referent**		**0.028**
C/T	101 (0.47)	217 (0.43)	1.309 (0.9295 –1.843)	0.123	1.430 (0.981–2.085)	0.063	
T/T	28 (0.13)	46 (0.09)	**1.712 (1.006–2.911)**	**0.0458**	**1.360 (1.011–1.828)**	**0.041**	
C/T + T/T	129 (0.60)	263 (0.52)	1.379 (0.9964–1.909)	0.0522	**1.423 (1.025–1.977)**	**0.035**	
MAF^c^	0.37	0.31	**1.303 (1.027–1.653)**	**0.029**	**1.373 (1.056–1.784)**	**0.018**	

Sex							
**Male**							
C/C	49 (0.35)	158 (0.48)	**Referent**	–	Referent	–	**0.035**
C/T	68 (0.49)	139 (0.43)	**1.577 (1.024–2.431)**	**0.0381**	**1.640 (1.059–2.540)**	**0.026**	
T/T	22 (0.16)	30 (0.9)	**2.365 (1.251–4.470)**	**0.0071**	**1.554 (1.124–2.147)**	**0.007**	
C/T + T/T	90 (0.65)	169 (0.52)	**1.717 (1.139–2.588)**	**0.0094**	**1.789 (1.181–2.712)**	**0.001**	
MAF^d^	0.40	0.30	**1.543 (1.152–2.066)**	**0.0035**	**1.610 (1.169–2.216)**	**0.003**	
**Female**							
C/C	36 (0.48)	81 (0.46)	Referent	–	Referent	–	0.747
C/T	33 (0.44)	78 (0.44)	0.952 (0.5407–1.676)	0.864	0.958 (0.539–1.702)	0.896	
T/T	6 (0.08)	16 (0.09)	0.844 (0.3051–2.334)	0.743	0.931 (0.520–1.667)	0.807	
C/T + T/T	39 (0.52)	94 (0.54)	0.934 (0.5430–1.605)	0.803	0.935 (0.540–1.620)	0.809	
MAF^c^	0.30	0.31	0.935 (0.617–1.417)	0.752	0.973 (0.611–1.551)	0.909	

Significant results are highlighted in bold font.

^a^χ^2^. ^b^ORs were adjusted by age, BMI and presence of diabetes. ^c^Minor allele frequency.

The division of the patients based on sex revealed a significant contribution of rs67085638 to GC in the male patients (*p*_trend_ = 0.035). For T/T versus C/C, the adjusted OR was 1.554 (95% CI 1.124–2.147, *p* = 0.007); for C/T versus C/C, the adjusted OR was 1.640 (95% CI 1.059–2.540, *p* = 0.026); for C/T + T/T versus C/C, the adjusted OR was 1.789 (95% CI 1.181–2.712, *p* = 0.001); and for T versus C, the adjusted OR was 1.610 (95% CI 1.169–2.216, *p* = 0.003). However, there was no contribution of rs67085638 to GC in the female patients (*p*_trend_ = 0.747). For T/T versus C/C, the adjusted OR was 0.931 (95% CI 0.520–1.667, *p* = 0.807); for C/T versus C/C, the adjusted OR was 0.958 (95% CI 0.539–1.702, *p* = 0.896); for C/T + T/T versus C/C, the adjusted OR was 0.935 (95% CI 0.540–1.620, *p* = 0.809); and for T versus C, the adjusted OR was 0.973 (95% CI 0.611–1.551, *p* = 0.909).

### Prevalence of the *CCAT1* rs67085638 SNP among patients with cardia and non-cardia localization of GC

In the patients with cardia localization of GC, *p*_trend_ = 0.253, and for T/T versus C/C, the adjusted OR was 1.334 (95% CI 0.839–2.123, *p* = 0.222); for C/T versus C/C, the adjusted OR was 1.257 (95% CI 0.664–2.377, *p* = 0.481); for C/T + T/T versus C/C, the adjusted OR was 1.411 (95% CI 0.792–2.512, *p* = 0.241); and for T versus C, the adjusted OR was 1.279 (95% CI 0.820–1.993, *p* = 0.277).

In the patients with non-cardia localization of GC, we found a significant association of the rs67085638 SNP with GC, and the *p*-trend value calculated for the rs67085638 polymorphism was *p*_trend_ = 0.041. The logistic regression analysis demonstrated an association of the rs6983267 SNP with the C/T + T/T genotype as well as the T allele. For C/T + T/T versus C/C, the adjusted OR was 1.576 (95% CI 1.051–2.364, *p* = 0.028), and for T versus C, the adjusted OR was 1.404 (95% CI 1.047–1.881, *p* = 0.023). However, there was no significant association of the rs6983267 SNP with T/T versus C/C; the adjusted OR was 1.320 (95% CI 0.982–1.773, *p* = 0.065), and for C/T versus C/C, the adjusted OR was 1.252 (95% CI 0.922–1.985, *p* = 0.122).

### Prevalence of the *CCAT1* rs67085638 SNP in patients with diffuse, mixed and intestinal histological types of GC

There was no association of the rs67085638 polymorphism with diffuse, mixed and intestinal histological types of GC (Table 3). In the patients with diffuse histological types of GC, *p*_trend_ = 0.073, and for T/T versus C/C, the adjusted OR was 1.353 (95% CI 0.965–1.897, *p* = 0.078); for C/T versus C/C, the adjusted OR was 1.348 (95% CI 0.863–2.106, *p* = 0.189); for C/T + T/T versus C/C, the adjusted OR was 1.440 (95% CI 0.943–2.199, *p* = 0.091); and for T versus C, the adjusted OR was 1.402 (95% CI 0.999–1.966, *p* = 0.049). In the patients with mixed histological types of GC, *p*_trend_ = 0.493, and for T/T versus C/C, the adjusted OR was 1.238 (95% CI 0.552–2.774, *p* = 0.603); for C/T versus C/C, the adjusted OR was 1.810 (95% CI 0.579–5.657, *p* = 0.306); for C/T + T/T versus C/C, the adjusted OR was 1.427 (95% CI 0.541–3.759, *p* = 0.471); and for T versus C, the adjusted OR was 1.267 (95% CI 0.632–2.540, *p* = 0.503). In the patients with intestinal histological types of GC, *p*_trend_ = 0.306, and for T/T versus C/C, the adjusted OR was 1.206 (95% CI 0.804–1.809, *p* = 0.363); for C/T versus C/C, the adjusted OR was 1.278 (95% CI 0.767–2.128, *p* = 0.345); for C/T + T/T versus C/C, the adjusted OR was 1.314 (95% CI 0.781–2.211, *p* = 0.303); and for T versus C, the adjusted OR was 1.172 (95% CI 0.795–1.727, *p* = 0.422).

**Table 3:** Prevalence of the *CCAT1* rs67085638 polymorphism among localization of GC and histological type of GC.

GC localization							
Genotype	Patients (frequency)	Controls (frequency)	Odds ratio (95%CI)	*p*^a^	Adjusted odds ratio (95%CI)^c^	*p*	*p*_trend_
**Cardia**							
C/C	22 (0.41)	239 (0.48)	Referent	–	Referent	–	0.253
C/T	25 (0.46)	217 (0.43)	1.252 (0.686–2.285)	0.464	1.257 (0.664–2.377)	0.481	
T/T	7 (0.13)	46 (0.09)	1.653 (0.667–4.096)	0.273	1.334 (0.839–2.123)	0.222	
C/T + T/T	32 (0.59)	263 (0.52)	1.322 (0.747–2.339)	0.337	1.411 (0.792–2.512)	0.241	
MAF^d^	0.36	0.31	1.271 (0.840–1.925)	0.256	1.279 (0.820–1.993)	0.277	
**Non-cardia**							
C/C	63 (0.39)	239 (0.48)	Referent	–	Referent	–	**0.041**
C/T	76 (0.48)	217 (0.43)	1.329 (0.908–1.945)	0.143	1.252 (0.922–1.985)	0.122	
T/T	21 (0.13)	46 (0.09)	1.732 (0.964–3.113)	0.064	1.320 (0.982–1.773)	0.065	
C/T + T/T	97 (0.61)	263 (0.52)	1.399 (0.974–2.010)	0.069	**1.576 (1.051–2.364)**	**0.028**	
MAF^d^	0.37	0.31	**1.314 (1.009–1.710)**	**0.0421**	**1.404 (1.047–1.881)**	**0.023**	

Histological type							
**Diffuse**							
C/C	44 (0.40)	239 (0.48)	Referent	–	Referent	–	0.073
C/T	52 (0.47)	217 (0.43)	1.302 (0.837–2.024)	0.241	1.348 (0.863–2.106)	0.189	
T/T	15 (0.13)	46 (0.09)	1.771 (0.910–3.447)	0.0893	1.353 (0.965–1.897)	0.078	
C/T + T/T	67(0.60)	263 (0.52)	1.384 (0.910–2.104)	0.128	1.440 (0.943–2.199)	0.091	
MAF^d^	0.37	0.31	1.317 (0.972–1.785)	0.0747	1.402 (0.999–1.966)	0.052	
**Mixed**							
C/C	7 (0.39)	239 (0.48)	Referent		Referent	–	0.493
C/T	9 (0.50)	217 (0.43)	1.416 (0.5184–3.868)	0.495	1.810 (0.579–5.657)	0.306	
T/T	2 (0.11)	46 (0.09)	1.484 (0.2987–7.376)	0.644^b^	1.238 (0.552–2.774)	0.603	
C/T + T/T	11 (0.61)	263 (0.52)	1.428 (0.5446–3.744)	0.467	1.427 (0.541–3.759)	0.471	
MAF^d^	0.36	0.31	1.271(0.636–2.543)	0.4703^b^	1.267 (0.632–2.540)	0.503	
**Intestinal**							
C/C	33 (0.42)	239 (0.48)	Referent	–	Referent	–	0.306
C/T	37 (0.47)	217 (0.43)	1.235 (0.746–2.044)	0.411	1.278 (0.767–2.128)	0.345	
T/T	9 (0.11)	46 (0.09)	1.417 (0.636–3.160)	0.392	1.206 (0.804–1.809)	0.363	
C/T + T/T	46 (0.58)	263 (0.52)	1.267 (0.784–2.048)	0.334	1.314 (0.781–2.211)	0.303	
MAF^d^	0.35	0.31	1.201(0.8432–1.711)	0.310	1.172 (0.795–1.727)	0.422	

The number of patients with undetermined histological type GC [(C/C-1 (0.17); C/T-3 (0.50): T/T-2 (0.33)] was too small; therefore, we did not apply statistical logistic regression.

Significant results are highlighted in bold font.

^a^χ^2^ or ^b^Fisher’s exact test. ^c^ORs were adjusted by age, BMI and presence of diabetes. ^d^Minor allele frequency.

### Prevalence of the *CCAT1* rs67085638 SNP in patients in various tumour stages of GC

We found an association of the rs67085638 polymorphism with T3 and T4 tumour stages in the patients with GC (Table 4). In the patients with T3 tumour stage GC, *p*_trend_ = 0.014, and for T/T versus C/C, the adjusted OR was 1.522 (95% CI 1.039–2.230, *p* = 0.031); for C/T versus C/C, the adjusted OR was 1.807 (95% CI 1.087–3.006, *p* = 0.022); for C/T + T/T versus C/C. The adjusted OR was 1.716 (95% CI 1.022–2.879, *p* = 0.041), and for T versus C, the adjusted OR was 1.565 (95% CI 1.116–2.194, *p* = 0.009). In patients with T4 tumour stage GC, *p*_trend_ = 0.032, and for C/T versus C/C, the adjusted OR was 2.190 (95% CI 1.205–3.979, *p* = 0.010); for C/T + T/T versus C/C, the adjusted OR was 2.164 (95% CI (1.215–3.855, *p* = 0.009); and for T versus C, the adjusted OR was 1.505 (95% CI 1.048–2.159, *p* = 0.027). However, we did not observe an association of rs67085638 with T4 tumour stage; for T/T versus C/C, the adjusted OR was 1.403 (95% CI 0.924–2.133, *p* = 0.111).

**Table 4:** Prevalence of the *CCAT1* rs67085638 polymorphism among various tumour stages and differentiation grades of GC.

Tumor stage							
Genotype	Patients (frequency)	Controls (frequency)	Odds ratio (95%CI)	*p*^a^	Adjusted odds ratio (95%CI)^c^	*p*	*p*_trend_
**T1**							
C|C	14 (0.58)	239 (0.48)	Referent	–	Referent	–	0.244
C|T	9 (0.37)	217 (0.43)	0.708 (0.300–1.669)	0.428	0.649 (0.233–1.805)	0.406	
T/T	1 (0.04)	46 (0.09)	0.371 (0.476–2.89)	0.481^b^	0.615 (0.219–1.727)	0.355	
C/T + T|T	10 (0.42)	263 (0.52)	0.649 (0.283–1.489)	0.304	0.655 (0.284–1.511)	0.320	
MAF^d^	0.23	0.31	0.669 (0337–1.328)	0.335^**b**^	0.512 (0.209–1.257)	0.144	
**T2**							
C|C	18 (0.56)	239 (0.48)	Referent	–	Referent	–	0.937
C|T	8 (0.25)	217 (0.43)	0.490 (0.209–1.149)	0.0945	0.653 (0.253–1.686)	0.378	
T/T	6 (0.19)	46 (0.09)	1.732 (0.652–4.599)	0.265	1.329 (0.813– 2.173)	0.254	
C/T + T|T	14 (0.44)	263 (0.52)	0.707 (0.344–1.452)	0.343	1.001 (0.457–2.211)	0.989	
MAF^d^	0.31	0.31	1.022 (0.593–1.764)	0.937	1.389 (0.771–2.504)	0.273	
**T3**							
C|C	29 (0.34)	239 (0.48)	Referent	–	Referent	–	**0.014**
C|T	45 (0.52)	217 (0.43)	**1.709 (1.035–2.823)**	**0.0348**	**1.807 (1.087–3.006)**	**0.022**	
T/T	12 (0.14)	46 (0.09)	**2.150 (1.022–4.521)**	**0.0399**	**1.522 (1.039–2.230)**	**0.031**	
C/T + T|T	57 (0.66)	263 (0.52)	**1.786 (1.105–2.887)**	**0.0169**	**1.716 (1.022–2.879)**	**0.041**	
MAF^d^	0.40	0.31	**1.507 (1.080–2.102)**	**0.0154**	**1.565 (1.116–2.194)**	**0.009**	
**T4**							
C|C	24 (0.33)	239 (0.48)	Referent	–	Referent	–	**0.032**
C|T	39 (0.54)	217 (0.43)	**1.790 (1.042–3.074)**	**0.0331**	**2.190 (1.205–3.979)**	**0.010**	
T/T	9 (0.13)	46 (0.09)	1.948 (0.8507–4.463)	0.1094	1.403 (0.924–2.133)	0.111	
C/T + T|T	48 (0.67)	263 (0.52)	**1.817 (1.080–3.059)**	**0.0230**	**2.164 (1.215–3.855)**	**0.009**	
MAF^d^	0.39	0.31	**1.784 (1.266–2.514)**	**0.0009**	**1.505 (1.048–2.159)**	**0.027**	

Histological grading							
**G1**							
C|C	2 (0.28)	239 (0.48)	Referent	–	Referent	–	0.328
C|T	4 (0.57)	217 (0.43)	2.203 (0.399–12.151)	0.422^b^	1.144 (0.158–8.279)	0.893	
T/T	1 (0.14)	46 (0.09)	2.598 (0.231–29.265)	0.415^b^	1.540 (0.451–5.260)	0.489	
C/T + T|T	5 (0.71)	263 (0.52)	2.272 (0.437–11.824)	0.455^b^	0.946 (0.131–6.835)	0.956	
MAF^d^	0.43	0.31	1.687 (0.5802–4.904	0.384^b^	1.659 (0.569–4.839)	0.354	
**G2**							
C|C	31 (0.47)	239 (0.48)	Referent	–	Referent	–	0.947
C|T	29 (0.44)	217 (0.43)	1.030 (0.6011–1.766)	0.0913	1.057 (0.614–1.818)	0.841	
T/T	6 (0.09)	46 (0.09)	1.006 (0.3969–2.548)	0.9906	1.013 (0.634–1.618)	0.957	
C/T + T|T	35 (0.53)	263 (0.52)	1.026 (0.6135–1.716)	0.9220	1.332 (0.754–2.354)	0.322	
MAF^d^	0.31	0.31	1.013 (0.685–1500)	0.947	1.034 (0.697–1.536)	0.866	
**G3**							
C|C	52 (0.37)	239 (0.48)	Referent	–	Referent	–	**0.009**
C|T	68 (0.48)	217 (0.43)	1.440 (0.960–2.160)	0.0767	1.494 (0.992–2.250)	0.054	
T/T	21 (0.15)	46 (0.09)	**2.098 (1.155–3.812)**	**0.0136**	**1.468 (1.084–1.990)**	**0.013**	
C/T + T|T	89 (0.63)	263 (0.52)	**1.555 (1.059–2.284)**	**0.0237**	**1.616 (1.096–2.383)**	**0.015**	
MAF^d^	0.39	0.31	**1.438 (1.093–1.892)**	**0.0092**	**1.438 (1.091–1.893)**	**0.009**	

Significant results are highlighted in bold font.

^a^χ^2^ or ^b^Fisher’s exact test. ^c^ORs were adjusted by age, BMI and presence of diabetes. ^d^Minor allele frequency.

We did not find a significant association of rs67085638 with T1 and T2 tumour stages of GC (Table 4). In the patients with T1 tumour stage GC, *p*_trend_ = 0.244, and for T/T versus C/C, the adjusted OR was 0.615 (95% CI 0.219–1.727, *p* = 0.355); for C/T versus C/C, the adjusted OR was 0.615 (95% CI 0.219–1.727, *p* = 0.406); for C/T + T/T versus C/C, the adjusted OR was 0.655 (95% CI 0.284–1.511, *p* = 0.320); and for T versus C, the adjusted OR was 0.512 (95% CI 0.209–1.257, *p* = 0.144). In the patients with T2 tumour stage GC, *p*_trend_ = 0.937, and for T/T versus C/C, the adjusted OR was 1.329 (95% CI 0.813– 2.173, *p* = 0.254); for C/T versus C/C, the adjusted OR was 0.653 (95% CI 0.253–1.686, *p* = 0.378); for C/T + T/T versus C/C, the adjusted OR was 1.001 (95% CI 0.457–2.211, *p* = 0.989); and for T versus C, the adjusted OR was 1.389 (95% CI 0.771–2.504, *p* = 0.273).

### Prevalence of the *CCAT1* rs67085638 SNP in patients with differentiation grading of GC

We observed an association of rs67085638 with G3 differentiation grading in the patients with GC (Table 4). In the patients with G3 differentiation grading of GC, *p*_trend_ = 0.009, and for T/T versus C/C, the adjusted OR was 1.468 (95% CI 1.084–1.990, *p* = 0.013); for C/T + T/T versus C/C, the adjusted OR was 1.616 (95% CI 1.096–2.383, *p* = 0.015); and for T versus C, the adjusted OR was 1.438 (95% CI 1.091–1.893, *p* = 0.009). However, we did not observe an association between rs67085638 and G3 differentiation grading; for C/T versus C/C, the adjusted OR was 1.494 (95% CI 0.992–2.250, *p* = 0.054). We did not find a significant association of rs67085638 with G1 and G2 differentiation grading of GC in patients with GC (Table 4). In the patients with G1 differentiation grading of GC, *p*_trend_ = 0.328, and for T/T versus C/C, the adjusted OR was 1.540 (95% CI 0.451–5.260, *p* = 0.489); for C/T versus C/C, the adjusted OR was 1.144 (95% CI 0.158–8.279, *p* = 0.893); for C/T + T/T versus C/C, the adjusted OR was 0.946 (95% CI 0.131–6.835, *p* = 0.956); and for T versus C, the adjusted OR was 1.659 (95% CI 0.569–4.839, *p* = 0.354). In the patients with G2 differentiation grading of GC, we found that *p*_trend_ = 0.947, and for T/T versus C/C, the adjusted OR was 1.013 (95% CI 0.634–1.618, *p* = 0.957); for C/T versus C/C, the adjusted OR was 1.057 (95% CI 0.614–1.818, *p* = 0.841); for C/T + T/T versus C/C, the adjusted OR was 1.332 (95% CI 0.754–2.354, *p* = 0.322); and for T versus C, the adjusted OR was 1.034 (95% CI 0.697–1.536, *p* = 0.866).

### Prevalence of the *CCAT1* rs67085638 SNP in patients with various lymph node and GC metastasis stages

We found a significant association of rs67085638 with N3 lymph node metastasis in GC (Table 5). In the patients with N3 lymph node metastasis of GC, *p*_trend_ = 0.0005, and for T/T versus C/C, the adjusted OR was 9.134 (95% CI 1.250–2.929, *p* = 0.003); for C/T versus C/C, the adjusted OR was 3.081 (95% CI 1.492–6.362, *p* = 0.002); for C/T + T/T versus C/C, the adjusted OR was 2.703 (95% CI 1.462–4.997, *p* = 0.001); and for T versus C, the adjusted OR was 1.980 (95% CI 1.345–2.915, *p* = 0.001). However, we did not observe an association between rs67085638 and the patients with N0, N1 and N2 lymph node metastasis (Table 5). In the patients with N0 lymph node metastasis of GC, *p*_trend_ = 0.626, and for T/T versus C/C, the adjusted OR was 1.120 (95% CI 0.639–1.808, *p* = 0.642); for C/T versus C/C, the adjusted OR was 1.224 (95% CI 0.739–2.027, *p* = 0.431); for C/T + T/T versus C/C, the adjusted OR was 1.319 (95% CI 0.769–2.262, *p* = 0.314); and for T versus C, the adjusted OR was 1.192 (95% CI 0.798–1.781, *p* = 0.390). In the patients with N1 lymph node metastasis, *p*_trend_ = 0.909, and for T/T versus C/C, the adjusted OR was 1.152 (95% CI 0.648–2.048, *p* = 0.629); for C/T versus C/C, the adjusted OR was 0.895 (95% CI 0.410–1.953, *p* = 0.780); for C/T + T/T versus C/C, the adjusted OR was 1.029 (95% CI 0.514–2.059, *p* = 0.935); and for T versus C, the adjusted OR was 1.082 (95% CI 0.639–1.830, *p* = 0.769). In the patients with N2 lymph node metastasis, *p*_trend_ = 0.475, and for T/T versus C/C, the adjusted OR was 1.324 (95% CI 0.810–2.167, *p* = 0.261); for C/T versus C/C, the adjusted OR was 0.998 (95% CI 0.448–2.222, *p* = 0.997); for C/T + T/T versus C/C, the adjusted OR was 1.118 (95% CI 0.562–2.225, *p* = 0.750); and for T versus C, the adjusted OR was 1.204 (95% CI 0.719–2.017, *p* = 0.480).

**Table 5:** Prevalence of the *CCAT1* rs67085638 polymorphism among various lymph nodes and stages of metastasis of GC.

Lymph node metastasis stage							
Genotype	Patients (frequency)	Controls (frequency)	Odds ratio (95%CI)	*p*^a^	Adjusted odds ratio (95%CI)^c^	*p*	*p*_trend_
**N0**							
C|C	34 (0.43)	239 (0.48)	Referent	–	Referent	–	0.626
C|T	37 (0.47)	217 (0.43)	1.199 (0.727–1.977)	0.478	1.224 (0.739–2.027)	0.431	
T/T	7 (0.09)	46 (0.09)	1.070 (0.447 –2.560)	0.880	1.120 (0.639–1.808)	0.642	
C/T + T|T	44 (0.56)	263 (0.52)	1.176 (0.727–1.902)	0.508	1.319 (0.769–2.262)	0.314	
MAF^d^	0.33	0.31	1.092 (0.762–1.567)	0.631	1.192 (0.798–1.781)	0.390	
**N1**							
C|C	17 (0.48)	239 (0.48)	Referent	–	Referent	–	0.909
C|T	14 (0.40)	217 (0.43)	0.907 (0.437–1.88)	0.794	0.895 (0.410–1.953)	0.780	
T/T	4 (0.11)	46 (0.09)	1.223 (0.393–3.801)	0.759^b^	1.152 (0.648–2.048)	0.629	
C/T + T|T	18 (0.51)	263 (0.52)	0.962 (0.485–1.910)	0.912	1.029 (0.514–2.059)	0.935	
MAF^d^	0.31	0.31	1.031 (0.612–1.738)	0.894^b^	1.082 (0.639–1.830)	0.769	
**N2**							
C|C	19 (0.45)	239 (0.48)	**Referent**	–	**Referent**	**–**	**0.475**
C|T	17 (0.40)	217 (0.43)	0.985 (0.499–1.945)	0.966	0.998 (0.448–2.222)	0.997	
T/T	6 (0.14)	46 (0.09)	1.641 (0.622–4.33)	0.313	1.324 (0.810–2.167)	0.261	
C/T + T|T	23 (0.55)	263 (0.52)	1.100 (0.584–2.071)	0.768	1.118 (0.562–2.225)	0.750	
MAF^d^	0.34	0.31	1.186 (0.743–1.896)	0.476	1.204 (0.719–2.017)	0.480	
**N3**							
C|C	15 (0.25)	239 (0.48)	Referent	–	Referent	–	**0.0005**
C|T	33 (0.56)	217 (0.43)	**2.423 (1.281–4.584)**	**0.0053**	**3.081 (1.492–6.362)**	**0.002**	
T/T	11 (0.19)	46 (0.09)	**3.810 (1.645–8.824)**	**0.001**	**9.134 (1.250–2.929)**	**0.003**	
C/T + T|T	44 (0.74)	263 (0.52)	**2.666 (1.446–4.915)**	**0.0012**	**2.703 (1.462–4.997)**	**0.001**	
MAF^d^	0.47	0.31	**1.964 (1.335–2.88)**	**0.0005**	**1.980 (1.345–2.915)**	**0.001**	

Metastasis stage							
**M0**							
C|C	76 (0.39)	239 (0.48)	Referent	–	Referent	–	**0.027**
C|T	90 (0.47)	217 (0.43)	1.304 (0.9132–1.863)	0.1435	1.348 (0.940–1.934)	0.104	
T/T	26 (0.14)	46 (0.09)	**1.777 (1.030–3.068)**	**0.0373**	**1.349 (1.023–1.778)**	**0.033**	
C/T + T|T	116 (0.60)	263 (0.52)	1.387 (0.989–1.945)	0.0575	**1.438 (1.021– 2.025)**	**0.037**	
MAF^d^	0.37	0.31	**1.320 (1.031–1.689)**	**0.0273**	**1.357 (1.033–1.782)**	**0.028**	
**M1**							
C|C	9 (0.41)	239 (0.48)	Referent	–	Referent	–	0.638
C|T	11 (0.50)	217 (0.43)	1.346 (0.547–3.311)	0.516	1.175 (0.608–5.060)	0.297	
T/T	2 (0.09)	46 (0.09)	1.155 (0.242–5.520)	0.695	1.019 (0.458–2.267)	0.962	
C/T + T|T	13 (0.59)	263 (0.52)	1.313 (0.5510–3.127)	0.538	1.729 (0.624–4.794)	0.291	
MAF^d^	0.34	0.31	1.163 (0.615–2.201)	0.621^b^	1.431 (0.703–2.913)	0.322	

Significant results are highlighted in bold font.

^a^χ^2^ or ^b^Fisher’s exact test. ^c^ORs were adjusted by age, BMI and presence of diabetes. ^d^Minor allele frequency.

We also found a contribution of the rs67085638 SNP to the M0 metastasis stage of GC (Table 5). In the patients with M0 metastasis stage GC, we observed *p*_trend_ = 0.027, and for T/T versus C/C, the adjusted OR was 1.349 (95% CI 1.023–1.778, *p* = 0.033); for C/T + T/T versus C/C, the adjusted OR was 1.438 (95% CI 1.021– 2.025, *p* = 0.037); and for T versus C, the adjusted OR was 1.357 (95% CI 1.033–1.782, *p* = 0.028). However, we did not observe an association of rs67085638 with M0 metastasis stage; for C/T versus C/C, the adjusted OR was 1.348 (95% CI 0.940–1.934, *p* = 0.104). We did not find a contribution of the rs67085638 SNP to the M1 metastasis stage of GC (Table 5). In the patients with M1 metastasis stage, we observed *p*_trend_ = 0.638, and for T/T versus C/C, the adjusted OR was 1.019 (95% CI 0.458–2.267, *p* = 0.962); for C/T versus C/C, the adjusted OR was 1.175 (95% CI 0.608–5.060, *p* = 0.297); for C/T + T/T versus C/C, the adjusted OR was 1.729 (95% CI 0.624–4.794, *p* = 0.291); and for T versus C, the adjusted OR was 1.431 (95% CI 0.703–2.913, *p* = 0.322).

### Influence of the rs67085638 polymorphism on BMI1 transcript levels in GC tissue and counterpart histopathologically confirmed disease-free margin tissue

**Figure 1:**
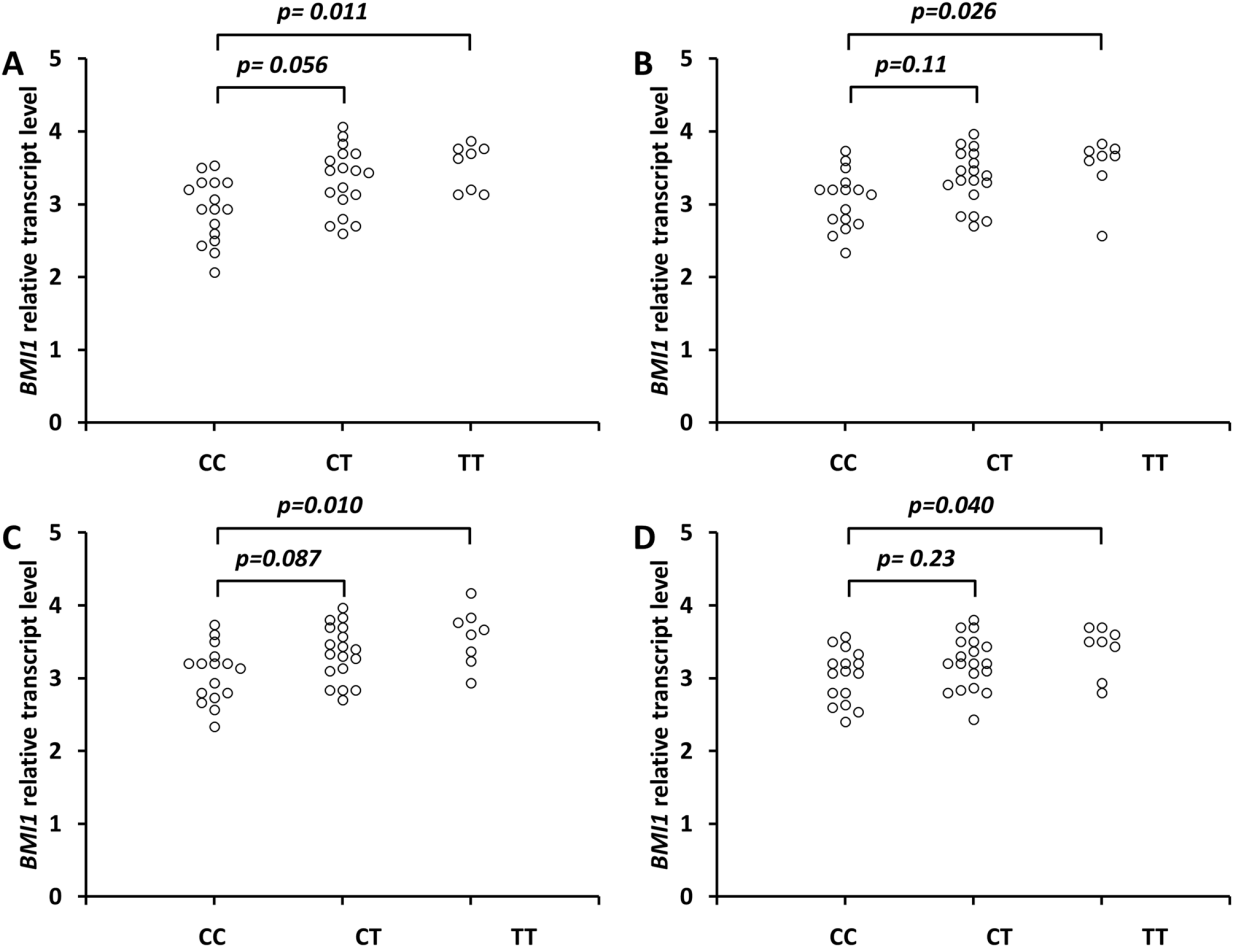
Effect of the *CCAT1* rs67085638 SNP on BMI1 transcript levels in primary GC tissue samples classified as grade G3 (**A**) and those classified as N3 lymph nodes (**C**) and distal counterpart surgical resection margins histopathologically confirmed disease-free for grade G3 (**B**) and N3 lymph nodes (**D**). Frozen tissue was homogenized, followed by total RNA isolation. Quantitative analyses of BMI1 transcript levels were performed by qRT-PCR using the SYBR Green I system (Supplementary Table 2). The quantity of BMI1 transcript levels in each sample was standardized by the geometric mean of references using HMBS and B2M cDNA levels. Kruskal–Wallis test with ^a^Dunn’s post hoc test (Supplementary Table 2).

We found significantly increased BMI1 transcript levels in the primary GC tissue classified as grade G3 (*p* = 0.011) and counterpart histopathologically confirmed disease-free margin tissue (*p* = 0.026, respectively) in the carriers of the T/T versus C/C genotypes but not for the carriers of the C/T versus C/C genotypes (*p* = 0.056, *p* = 0.11, respectively) (Fig. 1A, B). There was also a statistically significant increase in the BMI1 transcript levels in the primary GC tissue classified as lymph node metastasis N3 (*p* = 0.010) and counterpart histopathologically confirmed disease-free margin tissue (*p* = 0.040) in the carriers of the T/T versus C/C genotypes but not in the carriers of the C/T versus C/C genotypes (*p* = 0.087, *p* = 0.23, respectively) (Fig. 1C,D).

## Discussion

*CCAT1* and *CCAT2* are located in the 8q24.21 region, which is frequently amplified in colorectal cancer. In the 8q24 region, several complex molecular interactions are tissue-specific^29^ between CCAT1 and MYC, which regulate *CCAT1* and *MYC* gene expression^20,30–32^. *CCAT1* is situated inside a super‐enhancer region of the *MYC* promotor^32^. CCAT1-L protects against interactions between *MYC* and its enhancers, recruiting CCCTC‐binding factor or CTCF^20^. Moreover, CCAT1 lncRNA functions as a sponge for some tumour suppressor miRNAs to titrate the MYC protein^33^. In this context, MYC binds to the promoter of *CCAT1* and increases its transcription^16^.

Overexpression of CCAT1 has been observed in various malignancies compared to normal counterpart tissue, including GC^15^. CCAT1 displays oncogenic properties in various cancers, and in the cytoplasm or nucleus, it supports biological processes in cancer, such as proliferation, migration, cell cycle progression, apoptosis, and chemoresistance^15^. Moreover, CCAT1 positively correlated with the clinicopathological characteristics, progression and treatment outcomes of various cancers, including advanced TNM stage, vascular invasion, overall survival and recurrence-free survival, among others^15^.

In our studies, we observed a trend of rs67085638 SNP association in all patients with GC. rs67085638 was a strong risk factor for GC in male but not female patients. To date, the rs67085638 and rs7013433 polymorphisms have been demonstrated as risk factors for colorectal cancer^21^. However, we did not find an association between the rs7013433 polymorphism and either the occurrence of GC or the clinicopathological characteristics of GC (data not shown). The increased risk of rs67085638 in males can be due to the additive effect of environmental factors, including tobacco smoking, regular alcohol consumption, and limited consumption of fruits and vegetables, which are common in males in our country. Our observation is consistent with findings in other populations in which male sex is an important factor contributing to the development of GC^6,34^.

To date, the role of CCAT1 lncRNA in the development of GC has been intensively studied. CCAT1 was significantly elevated in primary GC tissue compared with normal gastric tissue and significantly correlated with the progression of GC and supports the proliferation and migration of GC cells^16–19^. A detailed study revealed that CCAT1 is involved in the negative regulation of the miR-219-1 and miR-490/hnRNPA1 axes, contributing to malignant transformation, progression and migration of GC cells^19,23,35^. CCAT1 expression was associated with larger gastric tumour size, lymphatic metastasis and advanced TNM stage^19^. CCAT1-L is also involved in the epithelial-mesenchymal transition and metastasis of gastric adenocarcinoma^18^.

We found that rs67085638 was associated with tumour stages T3 and T4, histological differentiation G3, lymph node metastasis stage N3 and metastasis stage M0. In addition, we determined that homozygous carriers of the CCAT1 rs67085638 T allele exhibited increased BMI1 transcript levels compared to homozygous carriers of the CCAT1 rs67085638 C allele from primary GC tissues as well as in histopathologically confirmed cancer-free margin tissue. Our findings suggest that the CCAT1 rs67085638 T allele elevating BMI1 expression might support increased rapid growth compared to lower-grade tumour cells and the expansion of cancer cells into the outer lining of the stomach or other organs and lymph node metastasis.

BMI1 is an oncogene and catalytic component of polycomb group proteins involved in epigenetic gene silencing. BMI1displays a critical function in tissue-specific regulation of gene expression and consequently several elementary cellular processes^36^. SNP rs6983267 has been shown to be located in the enhancer region of MYC, which plays a role in the regulation of CCAT1 expression^21^. Recently, it was demonstrated that the presence of the T allele of rs67085638 increased CCAT1 expression^22^. Moreover, CCAT1 expression correlated with BMI1 mRNA and protein levels in both GC cells in vitro and in vivo in murine tumour models^23^. CCAT1 knockdown repressed the proliferation and invasion of gallbladder cancer cells via miR-218-5p controlling BMI1 transcript translation^37^. In cigarette smoke extract-exposed human bronchial epithelial cells, CCAT1 negatively regulates miR-218, which modulates BMI1 expression and cell cycle progression^38^. BMI1 has been found to be a characteristic marker of poor prognosis in patients with breast cancer, nasopharyngeal carcinoma, oesophageal squamous cell carcinoma and gastric carcinoma^36,39,40^. Several studies have shown that BMI1 is associated with the progression, epithelial-mesenchymal transition and metastasis of GC^41–43^. The reduced expression of BMI1 inhibits epithelial-mesenchymal transition and spreading of melanoma cells^44^.

## Conclusion

Our study is the first to demonstrate that the *CCAT1* rs67085638 SNP is a risk factor for gastric carcinogenesis in Caucasian Polish individuals. We also found that homozygous carriers of CCAT1 rs67085638 T were associated with increased BMI1 transcript levels and increased rapid growth compared to lower-grade tumour cells, spread of cancer cells to neighbouring tissues and lymph node metastasis**.** However, our studies have some limitations. In patients with tumour stage T4, we observed the greatest contribution of C/T but not T/T to GC, which may be due to our relatively small group of patients with GC. This study should be repeated in other independent cohorts. Moreover, we need precisely explain the mechanism by which rs67085638 may regulate the transcription of *BMI1*.

## Supplementary Information

Supplementary Tables.
