# Supplementary material for: Long noncoding RNA CCAT1 rs67085638 SNP contribution to the progression of gastric cancer in a Polish population
Source: Sci Rep. 2021 Jul 28;11:15369. doi: 10.1038/s41598-021-94576-9 (PMC8319342; doi:10.1038/s41598-021-94576-9)
Supplement: Supplementary file 1 — Supplementary Tables. [file 41598_2021_94576_MOESM1_ESM.docx]

**Long noncoding RNA *CCAT1* rs67085638 SNP contribution to the progression of gastric cancer in a Polish population**

Tomasz Olesiński^1^, Anna Lutkowska^2^, Adam Balcerek^2^, Anna Sowińska^3^, Piotr Piotrowski^4^, Tomasz Trzeciak^5^, Tomasz Maj^1^, Piotr Hevelke^1^, Pawel, P. Jagodziński^2^

^1^Department of Oncological Gastroenterology, Maria Sklodowska-Curie National Research Institute of Oncology, Warsaw, Poland

^2^Department of Biochemistry and Molecular Biology, Poznań University of Medical Sciences Poznań, Poland

^3^Department of Computer Science and Statistics, Poznań University of Medical Sciences Poznań, Poland

^4^ Molecular Biology Department, National Geriatrics, Rheumatology and Rehabilitation Institute, Warsaw, Poland

^5^Department of Orthopedics and Traumatology, Poznan University of Medical Sciences, Poznan, Poland

Corresponding author: Dr. Paweł P. Jagodzinski, Department of Biochemistry and Molecular Biology, Poznań University of Medical Sciences, 6 Święcickiego St., 60-781 Poznań, Poland Tel: (48 61) 854 65 13, Fax: (48 61) 854 65 10, E-mail: pjagodzi@ump.edu.pl

Supplementary Table 1 Distribution of patients and controls between

different categories of body mass index (BMI)

|  |  |  |  |
| --- | --- | --- | --- |
| Categories of BMI (kg/m^2^) | 18.5 to < 24.9 | 25 to <30 | <30 |
| No. of controls | **232** | **152** | 118 |
| No. of patients | **114** | **58** | **42** |

BMI was calculated as weight divided by height squared (kg/m2) [25].

Diabetes was diagnosed in 36 patients with GC.

Supplementary Table 2 Experimental design, nucleic acid extraction, reverse transcription, qPCR target information

| **Item to check** | **Importance** |  |
| --- | --- | --- |
| **Experimental design** | | |
| Definition of experimental and control groups | E | The primary GC tissue samples were obtained from 42 patients with differentiation grade G3 and 42 patients with N3 lymph nodes. The control, which included distal counterpart surgical resection margin histopathologically confirmed disease-free tissue, was obtained from the corresponding primary GC tissue samples. Both primary and control tissue included patients with C/C, C/T and T/T genotypes. |
| Number within each group | E | GC differentiation grade G3 as well as GC with N3 lymph nodes included 42 patients with 16 patients with C/C, 18 patients with C/T, and 8 patients with T/T genotypes. The controls included the distal counterpart surgical resection margin histopathologically confirmed disease-free tissue corresponding to G/C differentiation grade G3 comprised of 16 patients with C/C, 18 patients with C/T, and 8 patients with T/T genotypes. GC with N3 lymph nodes comprised 16 patients with C/C, 18 patients with C/T, and 8 patients with T/T genotype. |
| Assay carried out by the core or investigator’s laboratory? | D | **N/A** |
| Acknowledgement of authors’ contributions | D | Agnieszka Mikuczewska |
| Sample | | |
| Description | E | GC and non-cancerous tissues |
| Volume/mass of sample processed | D |  |
| Microdissection or macrodissection | E | N/A |
| Processing procedure | E | N/A |
| If frozen, how and how quickly? | E | Frozen -20°C |
| If fixed, with what and how quickly? | E | N/A |
| Sample storage conditions and duration (especially for FFPE^b^ samples) | E | -20°C |
| Nucleic acid extraction | | |
| Procedure and/or instrumentation | E | Chomczyński and Sacchi method |
| Name of kit and details of any modifications | E | N/A |
| Source of additional reagents used | D |  |
| Details of DNase or RNase treatment | E | RNA samples were treated with DNase I |
| Contamination assessment (DNA or RNA) | E | qPCR amplification of genomic DNA fragment |
| Nucleic acid quantification | E | spectrophotometrically |
| Instrument and method | E | BioPhotometer Eppendorf AG (Hamburg, Germany) |
| Purity (A260/A280) | D |  |
| Yield | D |  |
| RNA integrity: method/instrument | E | agarose gel electrophoresis |
| RIN/RQI or Cq of 3_ and 5_ transcripts | E | N/A |
| Electrophoresis traces | D |  |
| Inhibition testing (Cq dilutions, spike, or other) | E | Cq dilutions of cDNA |
| Reverse transcription | | |
| Complete reaction conditions | E | 1 µg of Total RNA was dissolved in 8.25 µl H_2_O and added to mix (Total volume 5 µl) composed of 0.5 µl oligo dT (final concentration 2.5 µM), hexamers (final concentration 2.5 µM) and 4 µl dNTP (final concentration 2.5 µM) followed by 5 minute incubation in 70^o^C. After that reaction mixture was kept 1 minute on ice. In the second step the reaction mixture composed of 4 µl M-MLV buffer (final concentration x1), 2 µl DTT (final concentration 10 mM), 1 µl RNase OUT (final conc. 40U/μl) and 0.5 µl M-MLV (final conc. 100U/ul) was added to the first solution and then incubated 10 min in 25 ^o^C, 1 hour in 37 ^o^C, and 15 min in 75 ^o^C |
| Amount of RNA and reaction volume | E | 1 μg of RNA, reaction volume 20 μl |
| Priming oligonucleotide (if using GSP) and concentration | E | oligo d(T) (final conc. 2.5 µM) + hexamers (final conc. 2.5 µM) |
| Reverse transcriptase and concentration | E | Moloney Murine Leukaemia Virus (M-MLV) (final conc. 40U/μl) |
| Temperature and time | E | according to the manufacturer’s protocol |
| Manufacturer of reagents and catalogue numbers | D | Invitrogen, Life Technologies, (Carlsbad, CA), 28025013 |
| Cq^s^ with and without reverse transcription | D^c^ |  |
| Storage conditions of cDNA | D | -20°C |
| qPCR target information | | |
| Gene symbol | E | **BMI1** |
| Sequence accession number (ENST) | E | ENST00000376663.7 |
| Location of amplicon | D | 974-1137 |
| Amplicon length | E | 174 bp |
| In silico specificity screen (BLAST, and so on) | E | BLASTN 2.5.1+ (https://blast.ncbi.nlm.nih.gov/) |
| Pseudogenes, retropseudogenes, or other homologues | D |  |
| Sequence alignment | D | BLASTN 2.5.1+ (https://blast.ncbi.nlm.nih.gov/) |
| Secondary structure analysis of amplicon | D | Oligo 7.6 software (http://www.oligo.net/downloads.html) |
| Location of each primer by exon or intron (if applicable) | E | Forward primer – exon 7/8 junction  Reverse primer – exon 9 |
| What splice variants are targeted? | E | BMI1-201 |
| qPCR oligonucleotides | | |
| Primer sequences | E | BMI1 forward: ATCTAAGGAGGAGGTGAA  BMI1 reverse: TAGGCAATATCCATTAGTGTA  PBGD-forward: GCCAAGGACCAGGACATC  PBGD-reverse: TCAGGTACAGTTGCCCATC  B2M-forward: CACCCCCACTGAAAAAGATG  B2M-reverse: CCTCCATGATGCTGCTTACA |
| RTPrimerDB identification number | D | N/A |
| Probe sequences | D^d^ | N/A |
| Location and identity of any modifications | E | N/A |
| Manufacturer of oligonucleotides | D | Oligo.pl (Institute of Biochemistry and Biophysics Polish Academy of Sciences, Warsaw, Poland, http://oligo.ibb.waw.pl/) |
| Purification method | D | Salting-out method with ethanol precipitation |
| qPCR protocol | | |
| Complete reaction conditions | E | 5 μl Master Mix (2×conc.) + 1 μl MgCl_2_ (25 mM) + 1 μl F and R primer mix (5 μM each) + 1 μl cDNA + 2 μl H_2_O |
| Reaction volume and amount of cDNA/DNA | E | Reaction volume 10 μl (cDNA 1 μl) |
| Primer, (probe), Mg^2+^, and dNTP concentrations | E | Primer 0.5 μM, Mg^2+^ 2.5 mM, dNTP 0.8 mM |
| Polymerase identity and concentration | E | LightCycler 480 SYBR Green I Master Mix (Roche Diagnostics GmbH, Mannheim, Germany) |
| Buffer/kit identity and manufacturer | E | LightCycler 480 SYBR Green I Master Mix (Roche Diagnostics GmbH, Mannheim, Germany) |
| Exact chemical composition of the buffer | D | N/A |
| Additives (SYBR Green I, DMSO, and so forth) | E | SYBR Green I |
| Manufacturer of plates/tubes and catalogue number | D | Roche Diagnostics GmbH (Mannheim, Germany) |
| Complete thermocycling parameters | E | Preincubation: 10 min 95°C,  Amplification: 10 s 95°C, 10 s 60°C, 10 s 72°C with single fluorescence acquisition, 45 cycles, Ramp rate: 2.2°C (cooling) and 4.4°C (heating) Melting: 1 min 95°C, 1 min 40°C, 75-95°C with continuous fluorescence acquisition |
| Reaction setup (manual/robotic) | D | Manual |
| Manufacturer of qPCR instrument | E | Light Cycler®480 II Real-Time PCR System (Roche Diagnostics GmbH, Mannheim, Germany) |
| qPCR validation | | |
| Evidence of optimization (from gradients) | D | PCR with gradient temperature of annealing and electrophoresis |
| Specificity (gel, sequence, melt, or digest) | E | Melting curve |
| For SYBR Green I, Cq of the NTC | E | NTC with no amplification observed (Cq>40) |
| Calibration curves with slope and y intercept | E | The PCR amplification efficiency for target and reference cDNA was determined by different standard curves created by consecutive dilutions of the cDNA template mixture. |
| PCR efficiency calculated from slope | E | BMI1 E=2.0 |
| CIs for PCR efficiency or SE | D |  |
| r2 of calibration curve | E | R^2^ range from 0.99 to 1.0 |
| Linear dynamic range | E | cDNA dilution from 1 to 1/64 |
| Cq variation at LOD | E | N/A |
| CIs throughout range | D |  |
| Evidence for LOD | E | N/A |
| If multiplex, efficiency and LOD of each assay | E | N/A |
| Data analysis | | |
| qPCR analysis program (source, version) | E | Light Cycler®480 Software release 1.5.0 SP3 (Roche Diagnostics GmbH, Mannheim, Germany) |
| Method of Cq determination | E | Second derivative maximum method |
| Outlier identification and disposition | E | Peirce's criterion |
| Results for NTCs | E | No amplification observed for NTCs |
| Justification of number and choice of reference genes | E | Two reference genes: porphobilinogen deaminase (PBGD) and beta-2-microglobulin (B2M) |
| Description of normalization method | E | Relative quantification method with a calibrator. The calibrator was prepared as a cDNA mix from all cDNA samples and consecutive dilutions were used to create a standard curve. The quantity of **BMI1** transcript in each sample was standardized by the geometric mean of transcript levels. |
| Number and concordance of biological replicates | D |  |
| Number and stage (reverse transcription or qPCR) of technical replicates | E | Three |
| Repeatability (intraassay variation) | E | N/A |
| Reproducibility (interassay variation, CV) | D | N/A |
| Power analysis | D | N/A |
| Statistical methods for results significance | E | the Kruskal-Wallis test with Dunn's post hoc |
| Software (source, version) | E | Statistica version 10, 2011 (Stat Soft, Inc., Tulsa, USA) |
| Cq or raw data submission with RDML | D | N/A |

^a^All essential information (E) must be submitted with the manuscript. Desirable information (D) should be submitted if available. If primers are from RTPrimerDB, information on qPCR targets, oligonucleotides, protocols, and validation is available from that source.

^b^ FFPE, formalin-fixed, paraffin-embedded; RIN, RNA integrity number; RQI, RNA quality indicator; GSP, gene-specific priming; dNTP, deoxynucleoside triphosphate.

^c^Assessing the absence of DNA with a no–reverse transcription assay is essential when first extracting RNA. Once the sample has been validated as DNA free, inclusion of a no–reverse transcription control is desirable but no longer essential.

^d^Disclosure of the probe sequence is highly desirable and strongly encouraged; however, because not all vendors of commercial predesigned assays provide this information, it cannot be an essential requirement. Use of such assays is discouraged.
